# Short-Chain and Unsaturated Fatty Acids Increase Sequentially From the Lag Phase During Cold Growth of *Bacillus cereus*

**DOI:** 10.3389/fmicb.2021.694757

**Published:** 2021-07-22

**Authors:** Marina Français, Romain Bott, Claire Dargaignaratz, Christian Giniès, Frédéric Carlin, Véronique Broussolle, Christophe Nguyen-Thé

**Affiliations:** INRAE, Avignon Université, UMR SQPOV, Avignon, France

**Keywords:** fatty acids, *Bacillus cereus*, cold adaptation, lag phase, kinetic, short-chain fatty acids, unsaturated fatty acids

## Abstract

Fatty acids of two mesophilic and one psychrotrophic strains of the foodborne pathogen *Bacillus cereus* were analyzed by gas chromatography coupled to mass spectrometry during growth at cold (10 and 12°C) vs. optimal (30°C) temperatures and during the whole growth process (6–7 sampling times) from lag to stationary phase. In all these strains, a sequential change of fatty acids during cold growth was observed. Fatty acids were modified as soon as the end of lag, with an increase of the short-chain fatty acids (less than 15 carbons), particularly i13. These short-chain fatty acids then reached a maximum at the beginning of growth and eventually decreased to their initial level, suggesting their importance as a rapid cold adaptation mechanism for *B. cereus*. In a second step, an increase in Δ^5,10^ di-saturated fatty acids and in monounsaturated fatty acids in Δ^5^ position, at the expense of unsaturation in Δ^10^, started during exponential phase and continued until the end of stationary phase, suggesting a role in growth consolidation and survival at cold temperatures. Among these unsaturated fatty acids, those produced by unsaturation of n16 increased in the three strains, whereas other unsaturated fatty acids increased in some strains only. This study highlights the importance of kinetic analysis of fatty acids during cold adaptation.

## Introduction

*Bacillus cereus sensu lato* (*B. cereus sl*), a major cause of foodborne outbreaks in Europe (EFSA-BIOHAZ, 2016), is composed of closely related species (*B. cereus sensu stricto*, *Bacillus thuringiensis*, *Bacillus cytotoxicus*, *Bacillus weihenstephanensis*, *Bacillus wiedmanii*, *Bacillus toyonensis*, *Bacillus mycoides*, and *Bacillus pseudomycoides*) able to grow over a wide range of temperatures and in various environments (Guinebretiere et al., 2008). *B. cereus sl* is divided into seven phylogenetic groups, including the species mentioned earlier, according to their growth temperature range, from psychrotrophic (able to grow at 4°C) to nearly thermophilic groups (with a maximum growth temperature of 55°C). Adaptation to cold temperatures and the formation of highly resistant spores (Luu-Thi et al., 2014) make *B. cereus* an important hazard for heat-processed and refrigerated foods (EFSA, 2005). Inappropriate storage practices with exposure at abuse temperature or improper cooling, regularly observed at consumers’ home or in catering, for instance, are also opportunities for multiplication of mesophilic strains of *B. cereus sl* (ANSES, 2017).

*B. cereus* cold adaptation relies on multiple mechanisms (Brillard and Broussolle, 2012), such as glucose metabolism (Chung et al., 1976), over-synthesis of specific proteins such as cold acclimation proteins, including RNA-helicases (Broussolle et al., 2010; Pandiani et al., 2010, 2011), cold-shock proteins, DNA gyrases (Beckering et al., 2002), or activation of the CasKR two-component system (Diomande et al., 2014). A crucial mechanism for bacterial growth at cold temperatures is also change in fatty acid (FA) composition of the membrane to reduce its melting point, maintain fluidity, and hence exchanges with the extracellular environment (Haque and Russell, 2004; Russel, 2008; de Sarrau et al., 2012). The most common FAs of bacteria, including *B. cereus*, are straight-chain FAs, branched-chain FAs (BCFAs) in iso or anteiso position, saturated FAs (SFAs), and unsaturated FAs (UFAs) (Diomande et al., 2015c). In most bacteria, the melting point of membrane FAs at cold temperatures is lowered by the synthesis of short-chain FAs (SCFAs) (Kaneda, 1972), increase in UFAs relative abundance, and increase in anteiso/iso BCFAs ratio (Kaneda, 1977; Freese et al., 2008). Mesophilic *B. cereus* growth at relatively cold temperatures (12 or 15°C) was characterized by an increase in UFA and SCFA relative abundance (Haque and Russell, 2004; Brillard et al., 2010; de Sarrau et al., 2012; Chazarreta Cifre et al., 2013; Diomande et al., 2015a). However, the kinetic of these FA changes in *B. cereus* during growth is not known. In other bacterial species, a few studies investigated FA changes during the stationary phase (Annous et al., 1999) and during the whole growth at optimal and high temperatures (Lopes et al., 2019a,b), but none considered the whole cold adaptation and cold growth process, from lag to late stationary.

In particular, little is known on what happens during the early phase of growth at cold temperatures, when the bacteria has to adapt to its new growth environment. This hampers an accurate and robust estimation of growth and risk in refrigerated foods (Bertrand, 2019). This is particularly true for FA composition, which has so far been studied at a single phase of cold growth, either exponential or stationary. A dynamic view of FA modifications during cold temperatures adaptation is therefore lacking.

This work aims to examine FAs’ relative abundance during the whole growth process of *B. cereus*, from lag to stationary phase, to detect critical stages in changes of FA profiles, considering different *B. cereus* strains presenting *de facto* contrasted capacity to grow at cold temperatures. Consequently, we monitored the FA composition of two mesophilic and one psychrotrophic *B. cereus* strains, all along with growth, at 10, 12, and 30°C.

## Materials and Methods

### Bacterial Strains and Media

*B. cereus sl* strains American Type Culture Collection (ATCC) 14579^T^ and ATCC 10876 (both mesophilic strains, from *B. cereus* phylogenetic group IV, able to grow between 10 and 45°C) and MM3 (a psychrotrophic strain, from phylogenetic group II, growing between 7 and 40°C and recently affiliated to *B. wiedmanii*; Miller et al., 2016) were used. Strains were stored at –80°C in a 30% glycerol solution. For experimental purpose, *B. cereus* cells were grown in mAOAC made of synthetic broth AOAC (Wright and Mundy M334 broth, HiMedia Laboratories) at pH 7.0, sterilized by autoclaving at 121°C for 20 min and supplemented with a filter-sterilized glucose solution to a final concentration of 6.9 mM (Diomande et al., 2015a; Français et al., 2019).

### Growth Experiments

One purified colony of a 24-h culture was grown overnight at 30°C in mAOAC broth for each tested *B. cereus* strain. An aliquot of each of these cultures was diluted into 100 ml of mAOAC medium to obtain an A_600_ of 0.1. The inoculated medium was incubated at 30°C, under shaking at 200 rpm until an A_600_ of 0.5 and was used as inoculum for growth experiments.

Growth experiments were done at 30°C for warm temperatures and at 10 and 12°C for cold temperatures. Thirty degrees Celsius represented conditions close to the optimum for both the mesophilic and psychrotrophic strains (Diomande et al., 2015b). Twelve degrees Celsius represented a cold temperature that permitted a substantial growth of the mesophilic strains in the AOAC medium (Diomande et al., 2015a). Ten degrees Celsius was used for the psychrotrophic strain to have the same interval from the minimum growth temperature as for the mesophilic strains.

For each strain, the inoculum was 10-fold diluted in 500 ml of mAOAC. Cultures were then incubated in a thermostatically controlled incubator at the tested temperature (30, 12, or 10°C) under shaking at 200 rpm. Colony-forming unit (CFU) counts and A_600_ were regularly measured to monitor growth. CFU counts were obtained by plating serial dilutions on Luria Bertani (Biokar) agar plates incubated overnight at 30°C. Lag times were determined by fitting the growth curves to the Baranyi model using the Combase online tool.^1^ Additional growth was done in microtiter plates in a controlled temperature, automated optical density reader (Flx-Xenius XMA, Safas, Monaco). Each experiment was performed in triplicates with independently prepared bacterial cultures. Volumes of 40 ml of bacterial cultures were collected at regular time points to obtain samples at all phases of bacterial growth. Sampling times at cold (warm) temperatures were designated by Cn (Wn) with 0 ≤ *n* ≤ 7.

### Fatty Acid Analysis

Samples were successively centrifuged at 10,000 × *g* for 10 and 5 min. Supernatants were discarded, and pellets were washed with 30 ml of saline buffer followed by two centrifugations at 7,000 × *g* for 5 min. Transesterification of lipids to produce fatty acid methyl esters (FAMEs) and gas chromatography coupled to mass spectrometry analysis were done as previously described (de Sarrau et al., 2012; Ginies et al., 2016). FAME extracts were injected into a gas chromatography coupled to mass spectrometry system (Shimadzu QP 2010) equipped with an UBWax capillary column 30 m × 0.25 mm × 0.5 μm (Interchim, Montluçon, France). Each FAME was identified by its retention time, using the equivalent chain length method and its characteristic ions (Brillard et al., 2010; de Sarrau et al., 2012; Diomande et al., 2015b). FA composition was determined by internal normalization and calculated by the following equation:

$$\%FAME=\frac{A\,r\,e\,a\,F\,A\,M\,E}{T\,o\,t\,a\,l\,A\,r\,e\,a\,F\,A\,M\,E\,s}$$

### Statistical Analysis

A principal component analysis (PCA) was done for each strain with XLSTAT software (Addinsoft) using FAs’ relative abundance as quantitative variables and growth steps at each temperature as observations. An analysis of variance (ANOVA) was done for each strain considering growth temperatures (two levels, cold and warm) and growth step (seven levels) as factors, FAs relative abundance as quantitative variables, and their combinations. Comparison of mean abundance in each FA between each growth temperature–growth step combination was performed by Tukey’s honest significant difference test at 5% level.

## Results

### Growth of the Three *B. cereus* Strains at Cold and Warm Temperatures and Selection of Samples Analyzed for Fatty Acids

**FIGURE 1:**
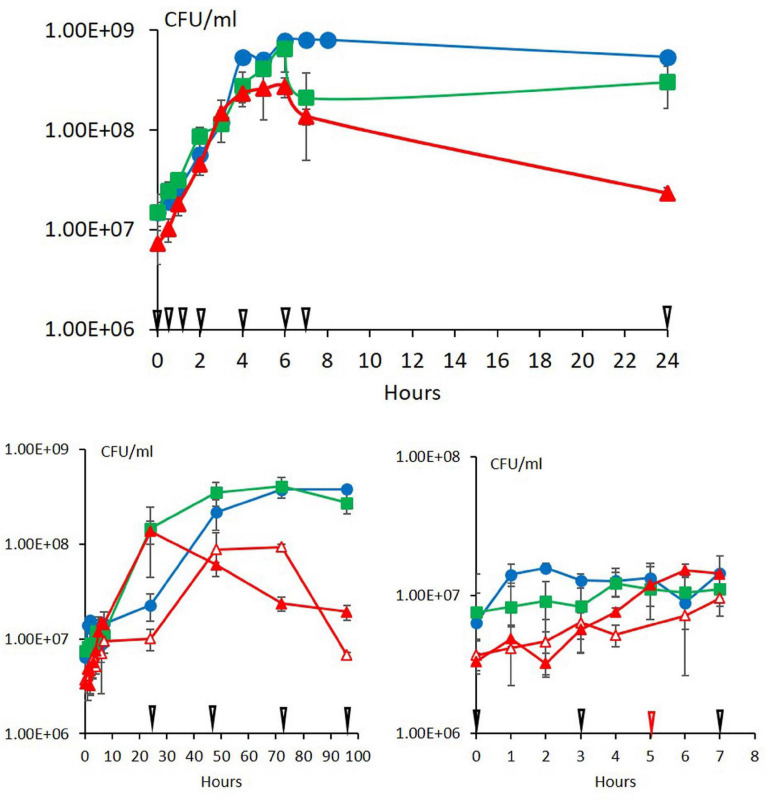
Growth of the three strains of *B. cereus* ATCC 10876 (green squares), ATCC 14579^T^ (blue circles), and MM3 (red triangles) at warm (upper panel) and cold (lower panels) temperatures. Warm temperature was 30°C for all strains. Cold temperatures were 12°C (close symbols) and 10°C for MM3 only (open symbols). Arrows indicate growth times at which cultures were sampled for fatty acid analysis (black: 0, 0.5, 1, 2, 4, 6, 7, and 24 h at 30°C, 0, 3, 7, 24, 48, 72, and 96 h at 10 and 12°C) and for MM3 at 10°C only (red: 5 h). At cold temperatures, growth during the first 7 h is detailed in the lower right-hand panel. Error bars represent standard deviation (*n* = 3).

At 30°C, the three strains ATCC 10876, ATCC 14579^T^, and MM3 had already increased in number as soon as 0.5 h, without noticeable lag time (Figure 1). For all strains, the first three sampling times for FA analysis at 0.5, 1, and 2 h corresponded to a phase of rapid growth, the fourth sampling time at 4 h corresponded to the end of rapid growth, the fifth to seventh sampling points (6, 7, and 24 h) to stationary phase.

At 12°C, MM3 was the first strain to initiate growth, then ATCC 10876 and ATCC 14579^T^ (Figure 1 for CFU and Supplementary Figure 1A for OD_600_). At 10°C, the growth of MM3 seemed to start immediately (lag time lower than 1 h, Figure 1). Lag time at 12°C could be estimated to 6 h for ATCC 10876 and to 18 h for ATCC 14579^T^ from CFU data. No estimation could be done for the lag time of MM3 at 12°C. Additional growth experiments with a high density of sampling times in microtiter plates with an automated optical reader confirmed this strain order ranking of increasing lag time at 12°C, from MM3 to ATCC 14579^T^ (Supplementary Figure 1B).

MM3 differed from the two other strains by a decline in numbers during the stationary phase, e.g., after a maximum reached at 24 h at 12°C. At 10°C, MM3 reached a maximum at 48 h, similarly to ATCC 14579^T^ and ATCC 10876 at 12°C. This is consistent with the fact that MM3 has a minimum growth temperature lower by 2°C compared with ATCC 10876 and ATCC 14579^T^ (Carlin et al., 2013). For this reason, in the present work, cold temperatures considered were 10°C for MM3 and 12°C for ATCC 10876 and ATCC 14579^T^. During growth at 12°C, the FA composition of MM3 changed according to similar patterns to 10°C (Supplementary Figure 2) and will not be presented in the subsequent sections.

### Global Fatty Acids Composition and Changes During Growth

Twenty-five FAs were identified and were the same for the three strains. Their chain length ranged from 12 to 18 carbons and consisted of linear, branched-chain iso, branched-chain anteiso, monosaturated, and di-saturated FAs. Considering all strains, growth temperatures, and times, the 10 most abundant FAs were nine FAs between 15 and 17 carbons long and the branched-chain i13 FA (Table 1). They contained linear, iso branched-chain, monounsaturated fatty in positions 5 or 10 and one di-UFA in positions 5 and 10. The same most abundant FAs (except i16) were also the ones with the highest range of variation observed over the complete experimental design (i.e., all temperatures and times tested).

**TABLE 1:** Fatty acids identified in *B. cereus* ordered by mean maximal relative abundance (left) and range of variation observed over all the strains (ATCC 10876, ATCC 14579^T^, and MM3), growth temperatures, and times considered in study.

	Mean* maximal abundance (%)		Range of variation**
n12	0.6	n12	0.5
n17	0.6	i18	0.6
i18	0.6	n17	0.6
n15	1.5	n15	1.5
i12	1.8	i12	1.5
n18:1	2.2	n18:1	2.1
i16:1 Δ^10^	4.9	i16:1 Δ^10^	4.7
a13	5.8	a13	4.8
n14	6.6	a15	5.2
n18	6.6	i14	5.2
a17	6.9	n14	5.9
a17:1 Δ^10^	7.4	a17	6.0
i17:1 Δ^10^	7.6	n18	6.1
i14	8.0	a17:1 Δ^10^	7.3
a15	8.7	i16	7.3
**i16:1 Δ^5^**	**8.8**	**i17:1 Δ^10^**	**7.4**
**i16**	**9.1**	**i16:1 Δ^5^**	**8.1**
**n16:1 Δ^5^**	**10.2**	**i13**	**8.4**
**i13**	**11.4**	**n16:1 Δ^5^**	**10.1**
**n16:2 Δ^5,10^**	**12.6**	**n16**	**12.5**
**i17**	**13.5**	**n16:2 Δ^5,10^**	**12.6**
**n16**	**14.0**	**i17**	**13.3**
**n16:1 Δ^10^**	**17.8**	**n16:1 Δ^10^**	**14.4**
**i17:1 Δ^5^**	**18.1**	**i17:1 Δ^5^**	**15.0**
**i15**	**31.8**	**i15**	**22.1**

*Mean of three replicate samples. **Difference between mean maximal abundance and mean minimal abundance among tested samples. Ten more abundant fatty acids or with the highest range are indicated in bold.

**FIGURE 2:**
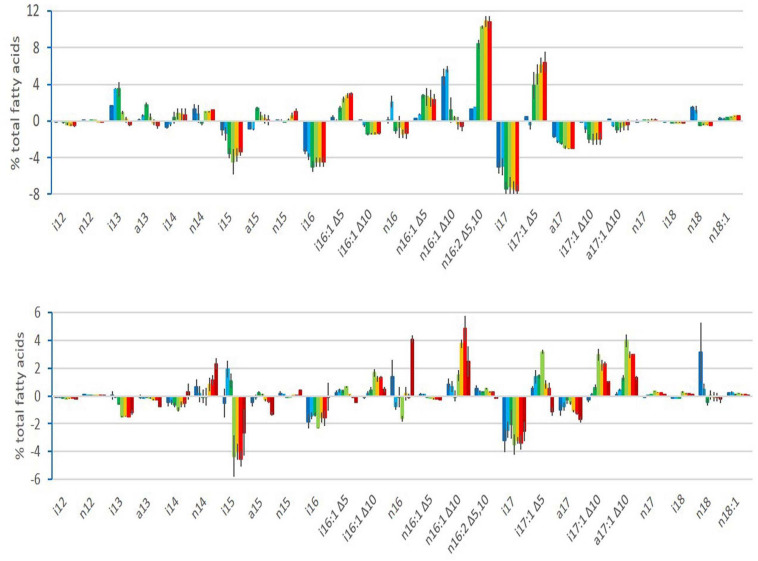
Changes in relative abundance (%) of fatty acids during growth of *B. cereus* strain ATCC 10876 at cold temperature (12°C, upper panel) and warm temperature (30°C, lower panel), relatively to the inoculum. Bars represent mean of three independent experiments and present for each growth time fatty acid relative abundance minus fatty acid relative abundance of inoculum. Lines over or below bars are standard deviations. For each fatty acid, growth times were, from left to right, 3, 7, 24, 48, 72, and 96 h at 12°C and 0.5, 1, 2, 4, 6, 7, and 24 h at 30°C. Colors ranged from dark blue for first sampling time to red for sixth (brown for the seventh at 30°C) sampling time.

Changes in the relative abundance of FAs were observed during growth at both cold and warm temperatures, as illustrated in Figure 2 for strain ATCC 10876 and for the two other strains, MM3 and ATCC 14579^T^, in Supplementary Figure 2. In Figure 2 and Supplementary Figure 2, FAs’ relative abundance is presented as an increase or decrease from the abundance in the inoculum. The FAs’ composition of the inoculum for the three strains is available in Supplementary Figure 3. For both cold and warm growth experiments, the inoculum was a mid-exponential culture at a warm temperature. Changes observed during growth at warm temperatures are therefore linked to growth phases only, whereas those occurring during growth at cold temperatures are linked to both cold adaptation and growth phases.

### Both Growth Temperature and Growth Phase Modified Fatty Acid Composition of *B. cereus*

**FIGURE 3:**
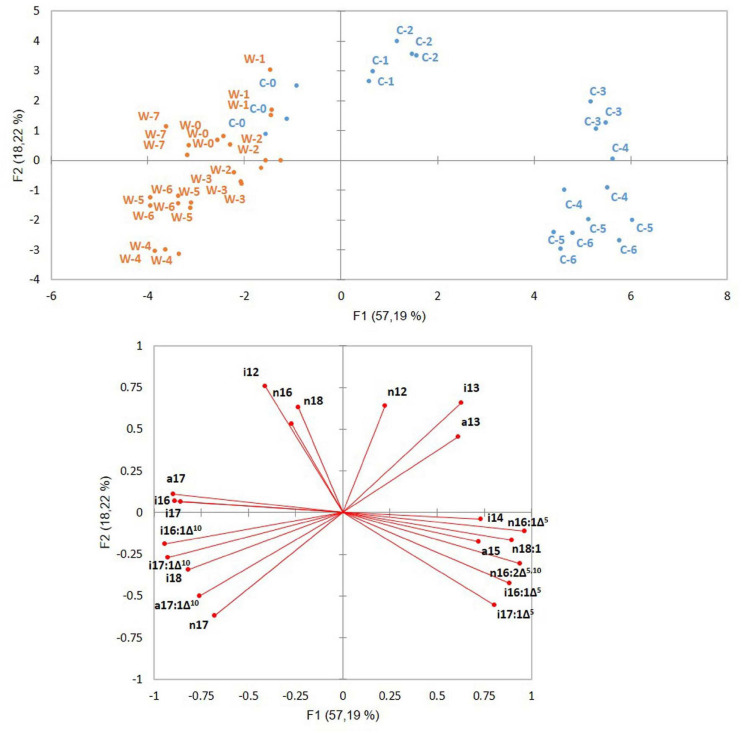
Principal component analysis of *B. cereus* ATCC 10876 samples grown at warm (W) and cold (C) temperatures. Samples were grown for 0, 3, 7, 24, 48, 72, and 96 h at cold (corresponding to samples C0 to C6, blue points) and 0, 0.5, 1, 2, 4, 6, 7, and 24 h at warm temperatures (corresponding to samples W0 to W 7, orange points). Inoculum at both cold (C0) and warm (W0) growth experiments were grown at a warm temperature. Distribution of samples (upper panel) and position of variables (lower panel). Variables were relative abundances of fatty acids.

The PCA of ATCC 10876 samples is shown in Figure 3. Samples grown at cold (12°C) and warm (30°C) temperatures were clearly separated along the first axis F1, which accounted for 57.2% of the variance. Time zero samples from warm and cold experiments clustered together with warm samples as both cold and warm growth experiments started from warm grown inocula. The second axis F2 (18.2%) of the variance discriminated samples according to the growth time. Samples at cold early growth separated from samples at late growth (growth times C1–C3 vs. C4–C6, corresponding to, respectively, the 3–24-h and 48–96-h growth periods). At warm temperature, growth time 4 (corresponding to 4 h) was the furthest from growth time 1 along axis F2, whereas the latest growth time (7, corresponding to 24 h) was close to time zero.

The variables (FAs’ relative abundances) contributing the most to the first axis in the direction of the cold grown samples were Δ^5^ mono-UFAs (particularly n16:1Δ^5^), the Δ^5,10^ di-UFA, and the BCFAs i14 and a15. In the opposite direction, on the side of warm grown, samples were located Δ^10^ mono-UFAs and the long BCFAs i17, a17, and i16. The short BCFAs i13 and a13 contributed to both axes on the side of early and cold growth samples, whereas n16, i12, and i15 contributed to both axes on the side of early and warm growth samples (Figure 3).

**FIGURE 4:**
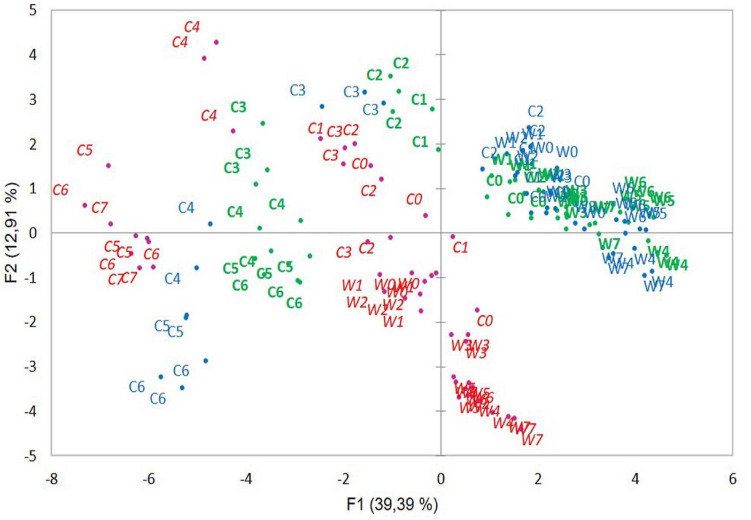
Distribution of samples in the principal component analysis of samples from three *B. cereus* strains MM3 (italic red), ATCC 14579^T^ (blue), and ATCC 10876 (bold green). Codes for growth temperatures and growth time are same as in Figure 3.

In PCA of the three strains together, all strain samples were distributed according to growth temperatures and growth periods (Figure 4) as shown for strain ATCC 10876 in Figure 3. ATCC 10876 and ATCC 14579^T^ samples grown at warm temperatures were distributed together, whereas those from MM3 stood clearly apart. At cold and late growth, ATCC 14579^T^ samples were located at more extreme positions of both axes than ATCC 10876.

### Few Fatty Acids Changed Similarly During Growth Among the Three Strains of *B. cereus*

For each strain, an ANOVA was done for each of the 25 FAs’ relative abundances as quantitative variables, considering the two factors, growth temperature and growth time.

For the three strains, growth temperature, growth time, and their interaction had a significant effect (*p* < 0.05) for most FAs, and for all FAs, at least one factor and/or the interaction had a significant effect (Supplementary Table 1).

For each FA, Tukey’s honest significant difference test at 5% level was used to compare all pairs of mean relative abundance for each growth temperature–growth time combination. This resulted in a classification of eight patterns (named ColdA, ColdB, ColdC, WarmA, WarmB, WarmC, ND, and NC) of changes in each FA abundance with temperature and time (Table 2). Some FAs were significantly more abundant at cold temperatures than at warm temperatures, at least at some stages of growth (patterns ColdA and ColdB in Table 2). FAs from ColdA significantly increased and then decreased, peaking in the middle of the growth experiment, while those from ColdB significantly increased during growth and reached a plateau. ColdC pattern was present only in MM3 and corresponded to FA with a slight but significant decrease at warm, vs. no changes at cold, temperature. Some FAs were associated with growth at warm temperature (WarmA, WarmB, and WarmC in Table 2) because they significantly increased during growth at warm temperature (WarmB and WarmC) and/or significantly decreased during growth at cold temperatures (WarmA and WarmC). Other FAs changed similarly at both warm and cold temperatures (Pattern ND) or showed no significant changes during growth or punctual changes with no clear trend (NC).

**TABLE 2:** Classification of fatty acids according to their patterns of changes during growth at cold and warm temperatures for three *B. cereus* strains ATCC 10876, ATCC 14579^T^, and MM3.

**Patterns of changes**	**Changes at cold temperature**	**Changes at warm temperature**	**ATCC 10867—12°C**	**ATCC 14579^T^—12°C**	**MM3—10°C**
ColdA	Increase followed by a decrease	Stable or decrease	**i13**, a13, a15, n12	i12, n12, **i13**, a13, a15	i12, **i13**, n14
ColdB	Increase to a plateau or steady increase	Stable or decrease	i14, n15, i16:1 Δ^5^, **n16:1 Δ^5^**, **n16:2 Δ^5,10^**, i17:1 Δ^5^, **n18:1**	i14, i16:1 Δ^5^, **n16:1 Δ^5^,** **n16:2 Δ^5,10^**, i17:1 Δ^5^, **n18:1**	a13, i14, a15, **n16:1 Δ^5^,** **n16:2 Δ^5,10^**, **n18:1**
ColdC	Stable	Decrease			i16:1 Δ^5^
WarmA	Decrease	Stable	i16, i17, a17	i16:1 Δ^10^, n16:1 Δ^10^**, n18, a17:1 Δ^10^	i16, i16:1 Δ^10^***, i17:1 Δ^10^
WarmB	Stable	Increase	n14, n16*, n16:1 Δ^10^*, n17	i16, **n16,** i17, i18	**n16**, a17, a17:1 Δ^10^
WarmC	Decrease	Increase	i16:1 Δ^10^, i17:1 Δ^10^, a17:1 Δ^10^, i18	i17:1 Δ^10^, a17	
ND	Decrease	Decrease	i12, **i15**	**i15**	**i15**
NC	No clear change	No clear change	n18	n14, n15, n17	n12, n15, n16:1 Δ^10^, i17, i17:1 Δ^5^, n17, i18, n18

Fatty acids with same patterns of changes for three strains are indicated in bold. *Early and transient increase at cold temperature, relative abundance similar at start and end of cold growth. **Early and transient increase at cold temperature, relative abundance lower at the end than at the start of cold growth. ***Decrease at the very end of the growth period at warm temperature. ND: No difference between behavior at cold and warm temperatures. NC: No clear changes at cold and warm temperatures.

Type ColdA corresponded to saturated SCFAs (<15 carbons), whereas ColdB-C corresponded to other SCFAs, mono-Δ^5^ UFAs, and the diΔ^5,10^ UFA. Most FAs were strain-specific, except i13 for ColdA and n16:1 Δ^5^, n16:2 Δ^5,10^, and n18:1 for ColdB (Table 2), which had the same pattern for all strains. These FAs were among the most abundant except n18-1 (Table 1). The warm temperature-associated FAs (WarmA and WarmB) were saturated FAs longer than 15 carbons (with n14 in ATCC 10876 being one exception) and Δ^10^ UFAs. Only n16 increased during growth at a warm temperature for all strains (categories WarmB).

Two FAs, i16:1 Δ^10^ and i17:1 Δ^10^ decreased at cold temperatures for all strains but did not have the same patterns of changes at a warm temperature for all strains. The FA i15 decreased during growth at both warm and cold temperatures for the three strains.

Strain MM3 differed from the two other strains by fewer FAs associated with warm temperature (n16:1 Δ^10^ and i17 with an NC pattern for MM3, instead of a WarmB or WarmC patterns) and by a smaller number of Δ^5^ mono-UFAs increasing at cold temperature (n16:1 Δ^5^ in ColdB only for MM3 against n16:1 Δ^5^, i16:1 Δ^5^, and i17:1 Δ^5^ for the two other strains).

### Changes in Fatty Acids Occurred at Different Growth Periods

**FIGURE 5:**
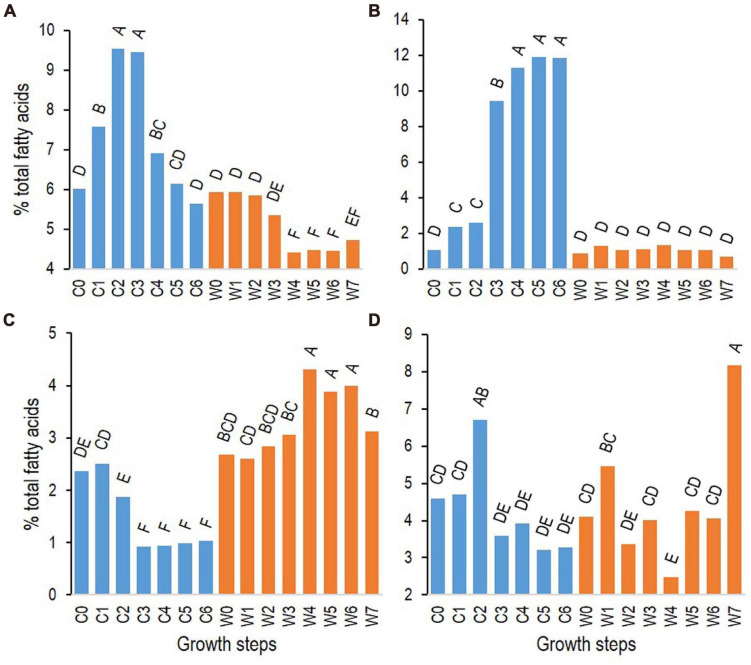
Changes of relative abundance at cold (12°C, C0–6, blue bars) and warm (30°C, W0–7, orange bars) temperatures of fatty acids with representative patterns of changes (see Table 2): i13 **(A)**, n16:2 Δ^5,10^ **(B)**, i16:1 Δ^10^ **(C)**, and n16 **(D)**. Results presented are from *B. cereus* strain ATCC 10876. Growth times C0–6 and W0–7 correspond to, respectively, 0, 3, 7, 24, 48, 72, and 96 h and 0, 0.5, 1, 2, 4, 6, 7, and 24 h. Results are mean of three independent experiments. In each panel, bars sharing same letter are not significantly different according to Tukey’s honest significant difference test at the 5% level.

**FIGURE 6:**
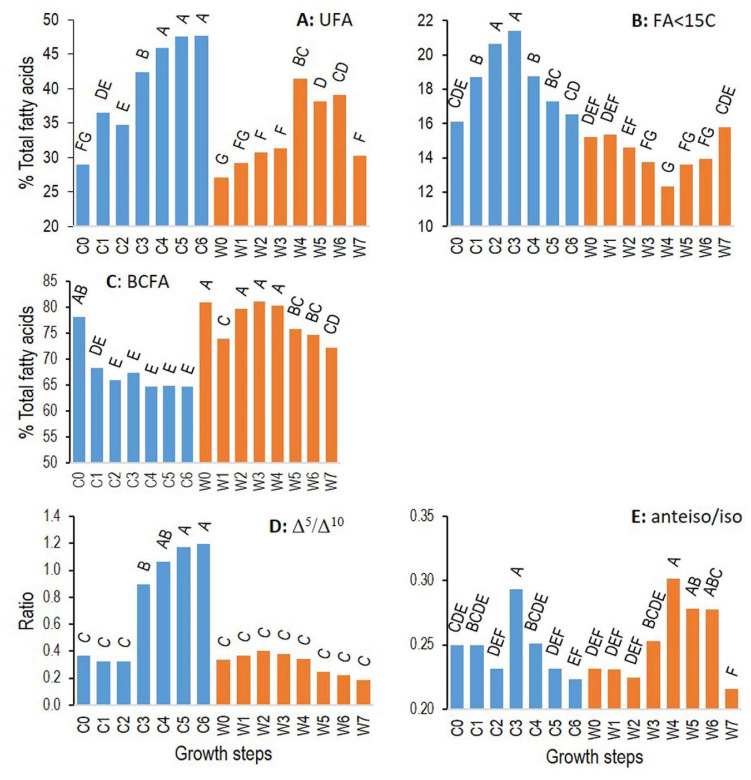
Changes for ATCC 10876 strain of *B. cereus* at cold (12°C, C0–6, blue bars) and warm (30°C, W0–7, orange bars) temperatures of relative abundance of total unsaturated fatty acids **(A)**, of fatty acids with less than 15 carbons **(B)**, of relative abundance of total branched-chain fatty acids **(C)**, of ratio Δ^5^ to Δ^10^ monounsaturated fatty acids **(D)**, and of ratio anteiso to iso branched-chain fatty acids **(E)**. Growth times C0–6 and W0–7 correspond to, respectively, 0, 3, 7, 24, 48, 72, and 96 h and 0, 0.5, 1, 2, 4, 6, 7, and 24 h. Results are mean of three independent experiments. In each panel, bars sharing same letter are not significantly different according to Tukey’ honest significant difference test at the 5% level.

Among FAs with similar patterns of changes among the three strains (Table 2), i13 increased rapidly during growth at cold temperatures, with a maximum relative abundance at growth step C2 (7 h). The FA n16:2 Δ^5,10^ increased later, at step C3 (24 h), reaching a maximum at growth step C4 (48 h) (Figure 5 for strain ATCC 10876 and Supplementary Figure 4 for other strains). At warm temperature, i16:1 Δ^10^ increased at step W4 (4 h) and decreased at the end of growth, whereas n16 increased at the very last step (24 h) (Figure 5 for strain ATCC 10876 and Supplementary Figure 4). It should be stressed that all replicate experiments were run independently, so some differences in the mean composition of the inoculum between cold and warm growth experiments were observed, although not significant, as shown in Figure 5. The same comment applied to Figure 6 in the next section.

### Changes of Fatty Acids Categories During Growth at Cold and Warm Temperatures

The relative abundance of FA categories was calculated, UFAs, FAs unsaturated in Δ^5^ and in Δ^10^ positions, FAs with less than 15 carbons (<C15), anteiso and iso branched FAs, and the ratio of Δ^5^–Δ^10^ and the ratio of anteiso to iso FAs. The two factors, growth temperatures and growth times, and their interaction had a significant effect (*p* < 0.05 according to the ANOVA) on all these variables except growth temperature for the “anteiso-to-iso” ratio (Supplementary Table 1).

Figure 6 presents the changes of these FA categories or their ratios for strain ATCC 10876. Total UFAs increased during growth at cold and warm temperatures, although the highest level reached at a cold temperature was higher than at a warm temperature. In addition, UFAs decreased at the last growth time that was analyzed at a warm temperature, i.e., at 24 h, but increased and reached a plateau at cold temperature. The position of the unsaturations strongly marked the differences between cold and warm temperatures. The ratio of Δ^5^–Δ^10^ increased sharply during growth at a cold temperature but remained constant at a warm temperature. The proportion of FAs with a short carbon chain, containing less than 15 carbons, increased to a peak at C3 (24 h) at a cold temperature and then decreased to the initial level, whereas it symmetrically decreased and increased at a warm temperature.

These two features of cold growth occurred at different growth times. SCFAs started to increase as soon as growth time C1 (3 h) and decrease after C3 (24 h), whereas the Δ^5^/Δ^10^ ratio remained constant until C2 (7 h), started to increase at C3 (24 h), and reached a plateau at C5 (72 h).

The ratio of anteiso to iso BCFAs increased during growth, to similar levels, at both cold and warm temperatures and then declined at the end of growth at both temperatures. The total relative abundance of BCFAs (anteiso plus iso) tended to decrease during growth at cold temperatures, resulting in a higher relative abundance in samples grown at warm temperatures until a decline at the last growth phase at a warm temperature.

Even if most FA considered individually had a strain-specific pattern of change during growth (Table 2), categories of FAs showed similar patterns among the three strains. Results presented in Figure 6 for ATCC 10876 were similar for ATCC 14579^T^ and MM3, however, with some variations in the extent and kinetics of changes at cold temperatures (Table 3 and Supplementary Figure 5). In particular, at step C0 (inoculum), the Δ^5^/Δ^10^ ratio was around 1 in MM3 strain against 0.3–0.4 in the strains ATCC 10876 and ATCC 14579^T^.

**TABLE 3:** Growth time at which changes in fatty acid categories were reached for *B. cereus* strains ATCC 10876, ATCC 14579^T^, and MM3 at cold and warm temperatures.

**Cold temperature**	**ATCC 10876—12°C**	**ATCC 14579^T^—12°C**	**MM3—10°C**
Maximum for short-chain (< C15) fatty acids	7 h	24 h	24 h
Maximum for unsaturated fatty acids (UFA)	48 h	96 h	48 h
Maximum for Δ^5^ to Δ^10^ ratio	72 h	96 h	96 h
Maximum for anteiso to iso ratio	24 h	48 h	72 h
**Warm temperature**	**ATCC 10876—30°C**	**ATCC 14579^T^—30°C**	**MM3—30°C**
Maximum for unsaturated fatty acids (UFA)	4 h	ns	6 h
Minimum for short-chain (<C15) fatty acids	4 h	ns	4 h
Δ^5^–Δ^10^ ratio	No change	No change	No change
Maximum for anteiso to iso ratio	4 h	4 h	4 h

*ns: Changes not significant.*

## Discussion

Changes in FA profiles occurred during growth at all temperatures, even at warm temperatures (30°C). As the inoculums were grown at 30°C in all experiments, changes at warm temperatures are reasonably due to the growth phase and not to adaptation to a new temperature. A few previous studies have shown FA changes in bacterial species other than *B. cereus*, during the stationary phase for *Pediococcus* sp. at 28°C (Annous et al., 1999) or during the whole growth for *Bacillus licheniformis* at 37 and 50°C (Lopes et al., 2019a,b). Globally, no significant changes at 30°C were observed during active growth, i.e., before 4 h (step W4) compared with the inoculum (W0). The inoculum was grown until the mid-exponential phase, and cells presumably did not need to change their FAs. Changes were observed at 4 h (step W4; Figures 5, 6 and Table 3), at a time corresponding to the end of growth entry in the stationary phase (Figure 1). These changes were characterized by increases in UFAs (notably some i16:1 Δ^10^), in anteiso/ratio, and by a decrease in SCFAs (Figures 5, 6). They presumably reflect membrane modifications linked to the shift from growth to survival during the stationary phase. However, the oldest cells tested (step W7 corresponding to 24 h at 30°C) were characterized by a fall of UFAs, of the anteiso-to-iso ratio, of BCFAs, and an increase of SCFA (Figures 5, 6). As a result, FA profiles of cells at step W7 were closer to the inoculum ones (W0), as indicated by the PCA (Figure 3). The increase in the relative abundance of n16, a straight chain and saturated FA, at step W7 (Figure 5), might result from the reduced synthesis of UFA and BCFAs in old cells. The increase of UFAs during growth at warm temperatures is surprising, as this is usually associated with cold growth.

UFAs have a lower melting point than their corresponding saturated FAs. An increase in the relative abundance of UFAs is a well-known mechanism to increase membrane fluidity at cold temperatures (Diomande et al., 2015c; de Carvalho and Caramujo, 2018). Previous studies reported higher monounsaturated proportions at cold than at optimal temperatures in *B. cereus* (Kaneda, 1972; Haque and Russell, 2004; de Sarrau et al., 2012; Chazarreta Cifre et al., 2013). In our work, mono-UFAs increased during growth at both cold and optimal temperatures, but their composition was markedly different, with a shift from Δ^10^ to Δ^5^ during growth at cold temperatures. An increase of Δ^5^ UFAs in *B. cereus* at 25°C compared with 37°C was previously observed (Chazarreta Cifre et al., 2013). However, Δ^5^ UFAs represented a minority of the UFAs at both temperatures, in contrast to our study done at much lower cold temperatures. To our knowledge, the shift between the proportions of unsaturation positions among mono-UFAs as a mechanism of cold adaptation in bacteria has not been previously reported. The melting point of mono-UFAs with unsaturation in an odd position (such as Δ^5^) is lower than that in an even position (such as Δ^10^) (Gunstone and Ismail, 1967; Knothe and Dunn, 2009). For cis-isomers of linear mono-UFAs of n-C18, the melting point difference between odd and even unsaturation is in the range of 10–15°C (Gunstone and Ismail, 1967; Knothe and Dunn, 2009). In comparison, the introduction of cis-monounsaturation in the n-C18 SFA decreases the melting point by 50–60°C (Jalal et al., 1982). The UFAs are diverse in *B. cereus*, including n-C16, i16, i17, and a17, and impacts of unsaturation positions on melting points of these FA are not documented yet. However, we assume that balance between Δ^10^ and Δ^5^ mono-UFAs allows a fine adjustment of membrane fluidity in relation to growth temperature, with a dominance of Δ^10^ unsaturations at warm temperature, less impacting membrane fluidity. A shift from Δ^10^ to Δ^5^ during growth at cold temperatures was observed for the three strains studied, stressing its importance for *B. cereus* cold adaptation. In addition, strain MM3, which is psychrotrophic, already had a higher Δ^5^–Δ^10^ ratio than the two mesophilic strains ATCC 10876 and ATCC 14579^T^ before growing at cold temperatures, which might contribute to its better cold adaptation. In the same line, a previous study also showed that mesophilic strains of *B. cereus* have a Δ^5^–Δ^10^ ratio lower than psychrotrophic ones when grown at optimal temperatures (Diomande et al., 2015b). Taken together, these results indicate the importance of the Δ^5^–Δ^10^ ratio in cold adaptation of *B. cereus*.

The increase in the di-UFA n16:2 Δ^5,10^ at cold temperatures for the three strains is consistent with previous findings on strain ATCC 14579^T^ (Chazarreta Cifre et al., 2013). *B. cereus* possesses two FA desaturases, DesA and DesB, both produced at cold temperatures and acting, respectively, on Δ^5^ and Δ^10^ positions (Chazarreta Cifre et al., 2013). In strain ATCC 14579^T^, Kristoffersen et al. (2012) found that at 30°C, *desB* was expressed at a much higher level than *desA*. At cold temperature, it was shown in the same strain that *desA* expression increased markedly (Diomande et al., 2016). These previous studies are consistent with our results showing a higher proportion of Δ^10^ unsaturation at a warm temperature and a higher proportion of Δ^5^ unsaturation at cold temperatures. In a mutant strain of ATCC 14579^T^ deleted for a sensor-regulator system and impaired for growth at cold temperature, *desA* was not induced at cold temperature, and Δ^5^ unsaturations did not increase at cold temperature (Diomande et al., 2015a). This further confirms the importance of DesA and Δ^5^ unsaturations in cold adaptation of *B. cereus*. However, the desaturase DesB presumably plays an important role together with DesA to both produce di-UFAs and adjust the Δ^5^–Δ^10^ ratio of mono-UFAs in the function of growth temperature. The only Δ^10^ FA with an early and transient increase at cold temperatures was n16 Δ^10^ (Table 2 and Figure 2). It then decreased concomitantly with the increase of n16:2 Δ^5,10^, indicating that DesA might desaturate the product of DesB action on n16. Diverse Δ^5^ mono-UFAs were observed over the three strains. However, among these mono-UFAs, only n16:1 Δ^5^ consistently increased at cold temperatures for the three strains. This suggests that n16 could be the main substrate for *B. cereus* desaturase at cold temperatures, with some variations among strains concerning the other FAs desaturated.

Both n16:2 Δ^5,10^ and Δ^5^ mono-UFAs started to increase between 7 and 24 h at cold temperatures (steps C2 and C3) for ATCC 10876 (Figures 5, 6) and between 24 and 48 h for ATCC 14579^T^ and MM3 (steps C3–C4 for ATCC 14579^T^ and C4–C5 for MM3, Supplementary Figures 4, 5). This corresponded to the exponential phase for the three strains (Figure 1). These FAs continued to increase or remained stable during the stationary phase. This suggests their importance for the growth and survival of *B. cereus* in cold conditions. The marked increase of Δ^5^ unsaturation during the exponential phase is consistent with previous results showing that *desA* was highly expressed during an exponential phase in ATCC 14579^T^ strain (Diomande et al., 2016).

Short-chain (<15C) FAs changed in opposite ways during growth at cold and optimal temperatures. They decreased at warm temperatures and increased at cold temperatures. At cold, they started to increase very early, between 3 and 7 h (steps C1–C2) for ATCC 10876 (Figure 6) and between 7 and 24 h for ATCC 14579^T^ and MM3 (steps C2–C3 for 14579^T^ and steps C3–C4 for MM3, Supplementary Figure 5). This corresponded to a phase where growth was not yet detectable for ATCC 10876 (i.e., lag phase) and has only just started (i.e., early growth) for the two other strains (Figure 1). Then, the SCFAs decreased after 24 h for ATCC 10876 and MM3 strains (steps C3 for 10876 and C4 for MM3) and after 48 h for ATCC 14579^T^ (step C4), corresponding in the three strains to the end of growth. SCFAs presumably have a role in the early steps of cold growth but not during the stationary phase. Although very little is known on what happens during the lag and early phase of bacterial growth (Bertrand, 2019), our work shows that an increase in SCFAs occurred during this early phase of *B. cereus* cold temperature adaptation. Among SCFAs, i13 increased particularly early during growth (Figure 5 and Supplementary Figure 4) and for the three strains studied. Previous studies showed the role of lipase in the increase of i13 at cold temperatures, as deletion of this lipase gene prevented the i13 increase and reduced *B. cereus* ATCC 14579^T^ growth at cold temperature (Brillard et al., 2010). Whether this lipase could modify preexisting FAs and contribute to a rapid reduction in the size of FAs during growth at cold temperatures is not known yet.

In conclusion, the kinetics view of FAs composition of three *B. cereus* strains during growth at cold and optimal temperature revealed a succession of two main changes during cold growth. First, cold adaptation starts with an increase in SCFAs before or during growth initiation and until the exponential phase, followed by an increase in di-unsaturated and in Δ^5^ UFAs at the expense of Δ^10^ UFAs during exponential phase and stationary phase.

## Data Availability Statement

The datasets presented in this study can be found in online repositories. The names of the repository/repositories and accession number(s) can be found below: Data are deposited in the open public repository “data INRAE” (https://data.inra.fr/) and accessible at: https://doi.org/10.15454/KCSN4H.

## Author Contributions

MF, VB, FC, CD, and CN-T contributed to the conception and the design of the study. MF, CD, and RB contributed to the acquisition of the data. MF, CD, RB, CG, and CN-T analyzed the data. MF, CG, and CN-T performed the statistical analysis. CN-T provided acquisition and project administration. MF wrote the first draft of the manuscript. All authors contributed to its revision, read and approved the submitted version.

## Conflict of Interest

The authors declare that the research was conducted in the absence of any commercial or financial relationships that could be construed as a potential conflict of interest.

## Footnotes

1 https://www.combase.cc/index.php/en/
