# Supplementary material for: Short-Chain and Unsaturated Fatty Acids Increase Sequentially From the Lag Phase During Cold Growth of Bacillus cereus
Source: Front Microbiol. 2021 Jul 22;12:694757. doi: 10.3389/fmicb.2021.694757 (PMC8339379; doi:10.3389/fmicb.2021.694757)
Supplement: Supplementary file 1 [file Data_Sheet_1.ZIP › Figure S2.pdf]

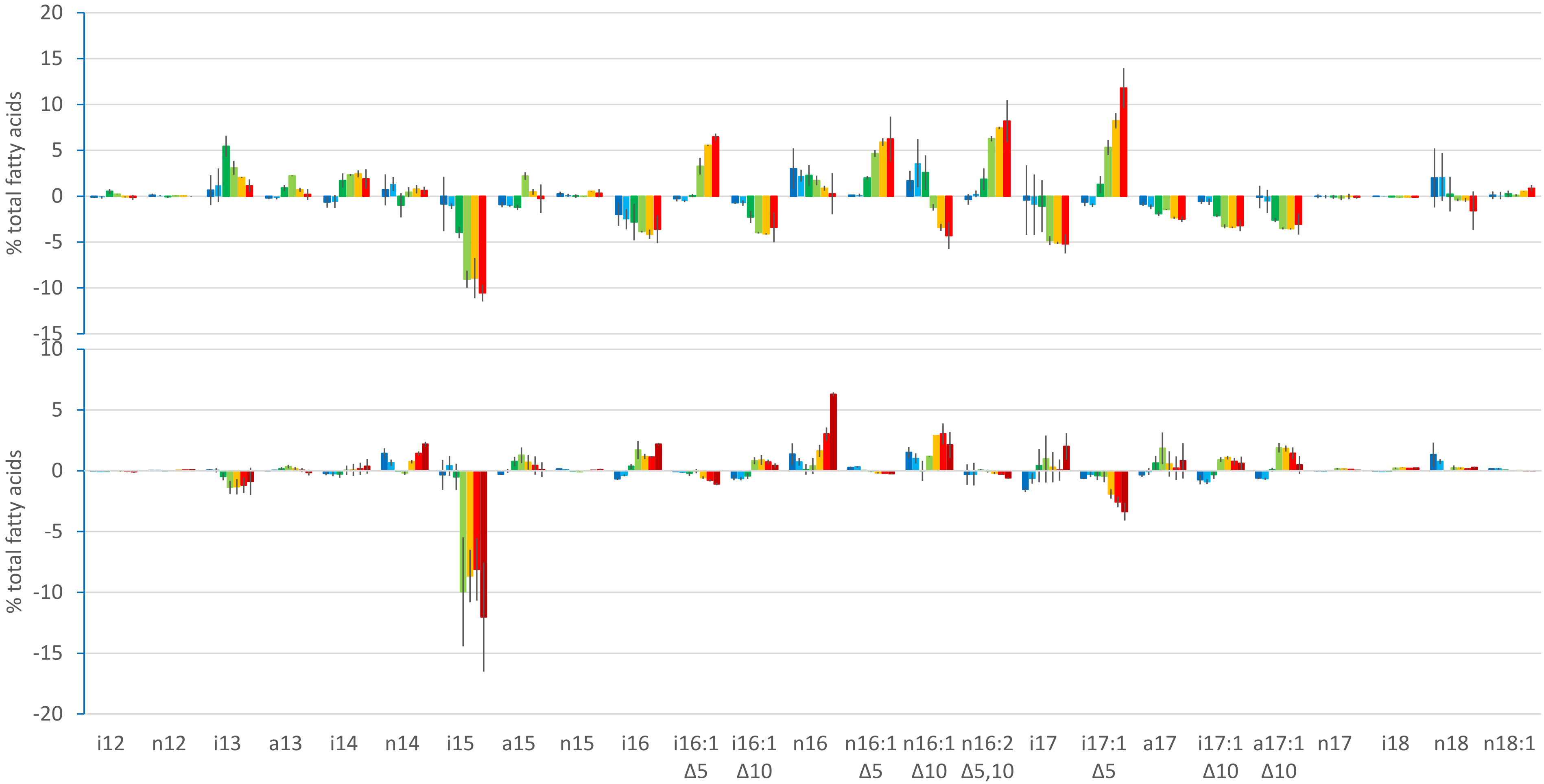

Figure S2a - Changes in relative abundance (%) of fatty acids during growth of *B. cereus* strain ATCC 14579^{T} at cold temperature (12 °C, upper panel) and warm temperature (30 °C, lower panel), relatively to the inoculum. Bars represent the mean of three independent experiments and present for each growth time fatty acid relative abundance minus fatty acid relative abundance of the inoculum. Lines over or below bars are standard deviations. Growth times were 3, 7, 24, 48, 72, 96 h at 12 °C and 0.5, 1, 2, 4, 6, 7, 24 h at 30 °C. Colors ranged from dark blue for the first sampling time to red for the 6th (brown for the 7th at 30 °C) sampling time.

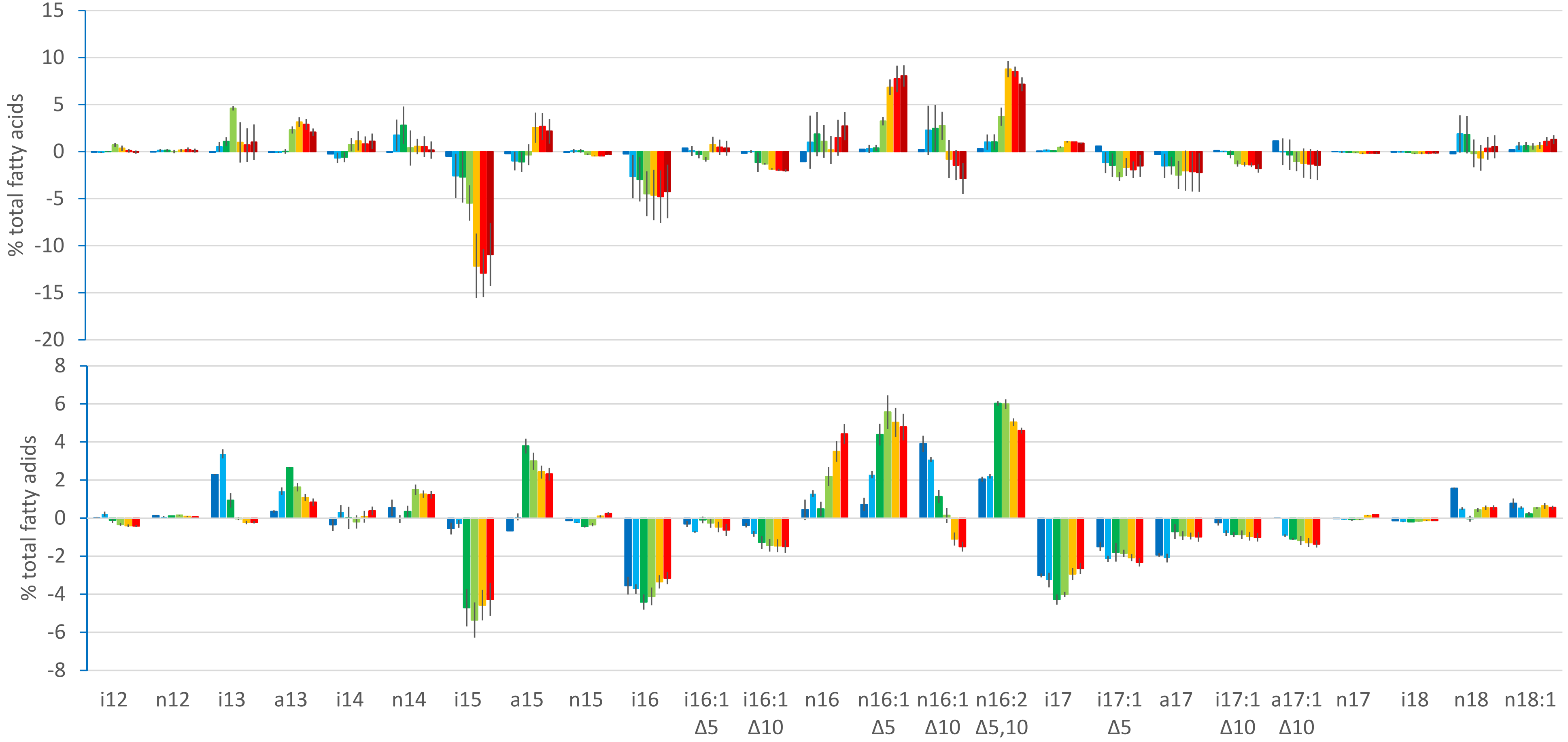

Figure S2b - Changes in relative abundance (%) of fatty acids during growth of *B. cereus* strain MM3 at cold temperatures of 10 °C (upper panel) and 12°C (lower panel), relatively to the inoculum. Bars represent the mean of three independent experiments and present for each growth time fatty acid relative abundance minus fatty acid relative abundance of the inoculum. Lines over or below bars are standard deviations. Growth times were 3, 7, 24, 48, 72, 96 h at 12 °C and 3, 5, 7, 24, 48, 72, 96 h at 30 °C. Colors ranged from dark blue for the first sampling time to red for the 6th (brown for the 7th at 10 °C) sampling time.

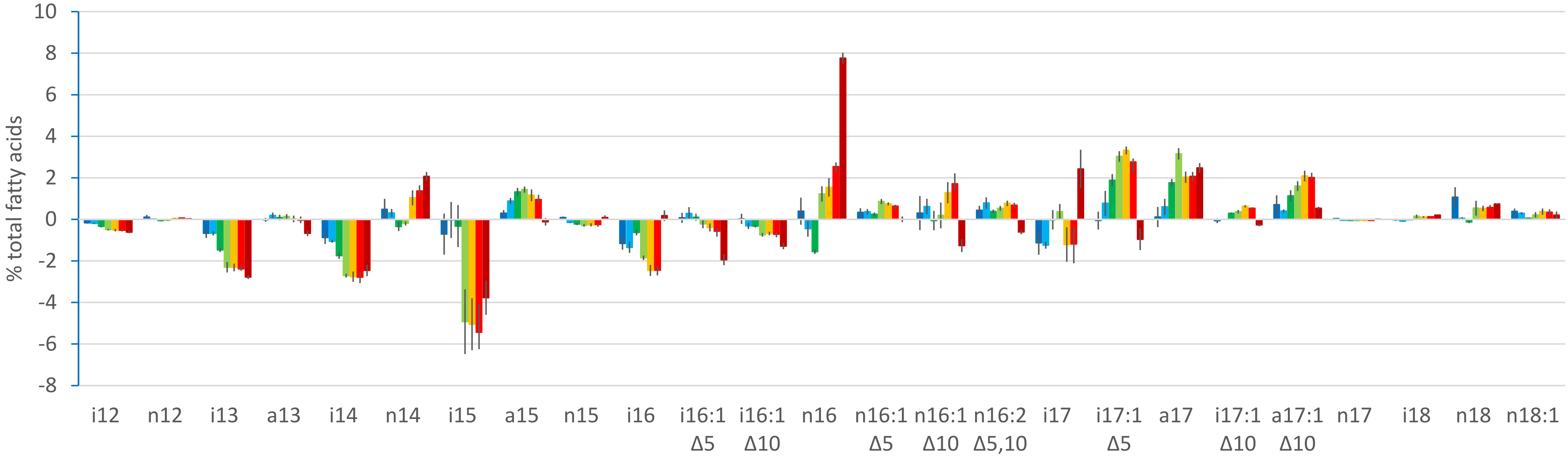

Figure S2c - Changes in relative abundance (%) of fatty acids during growth of *B. cereus* strain MM3 at warm temperature (30 °C), relatively to the inoculum. Bars represent the mean of three independent experiments and present for each growth time fatty acid relative abundance minus fatty acid relative abundance of the inoculum. Lines over or below bars are standard deviations. Growth times were 0.5, 1, 2, 4, 6, 7, 24 h. Colors ranged from dark blue for the first sampling time brown for the 7th sampling time.
