# Supplementary material for: Short-Chain and Unsaturated Fatty Acids Increase Sequentially From the Lag Phase During Cold Growth of Bacillus cereus
Source: Front Microbiol. 2021 Jul 22;12:694757. doi: 10.3389/fmicb.2021.694757 (PMC8339379; doi:10.3389/fmicb.2021.694757)
Supplement: Supplementary file 1 [file Data_Sheet_1.ZIP › Figure S3.pdf]

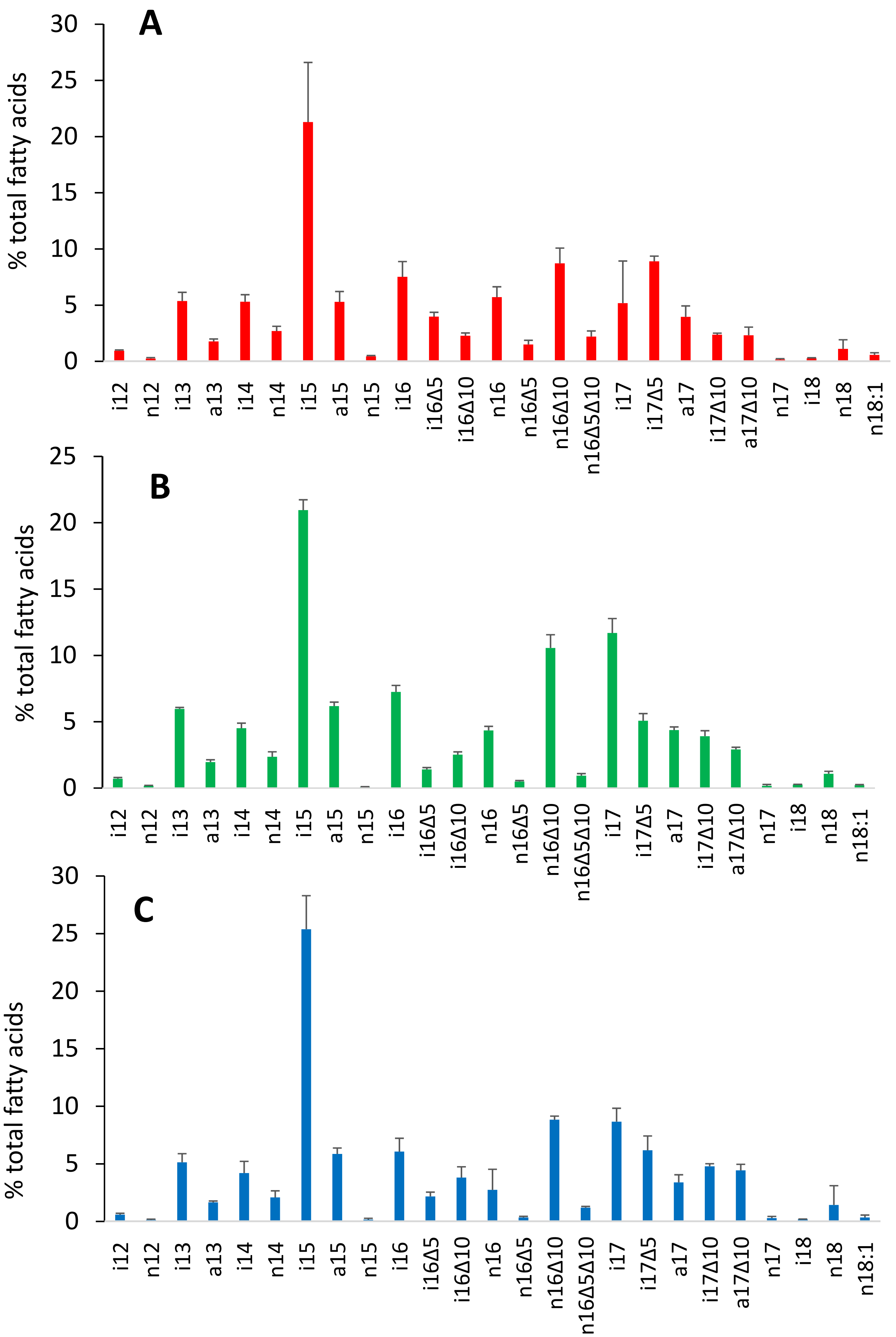

Figure S3 – Fatty acid profiles of the inoculum of strains MM3 (A), ATCC 10876 (B) and ATCC 14579^T (C), showing the relative abundance (in % of the total fatty acids) of all the identified fatty acids. All inoculums consisted of cells harvested at mid exponential phase at 30°C. Results are the mean with standard deviation of 6 (for ATCC 10876 and ATCC 14579^T) or 9 (for MM3) independent replicates
