# Supplementary material for: Short-Chain and Unsaturated Fatty Acids Increase Sequentially From the Lag Phase During Cold Growth of Bacillus cereus
Source: Front Microbiol. 2021 Jul 22;12:694757. doi: 10.3389/fmicb.2021.694757 (PMC8339379; doi:10.3389/fmicb.2021.694757)
Supplement: Supplementary file 1 [file Data_Sheet_1.ZIP › Figure S4.pdf]

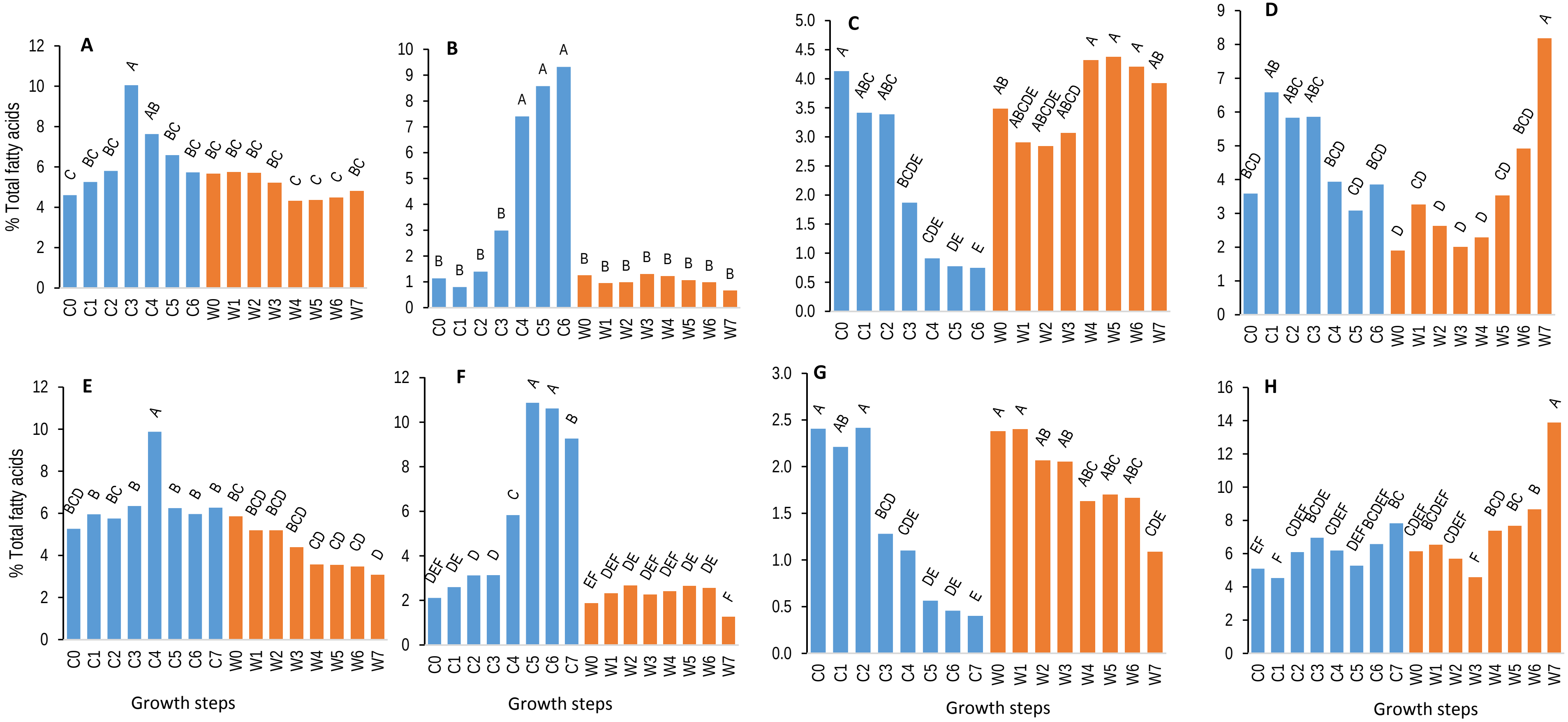

Figure S4 – Changes of relative abundance at cold (C0-7, blue bars) and warm (30 °C, W0-7, orange bars) temperatures of fatty acids with representative patterns of changes (see Table 2): i13 (A, E), n16:2 $\Delta^{5,10}$ (B, F), i16:1 Δ^{10} (C, G) and n16 (D, H). Results presented are from *B. cereus* strains ATCC 14579^T (A, B, C, D) and MM3 (E, F, G, H). Growth steps C0-6 for ATCC 14579^T correspond to 0, 3, 7, 24, 48, 72, 96 h at 12°C and C0-7 for MM3 to 0, 3, 5, 7, 24, 48, 72, 96 h at 10°C. W0-7 correspond for both strains to 0, 0.5, 1, 2, 4, 6, 7, 24 h. Results are the mean of three independent experiments. Bars sharing the same letter are not significantly different according to Tukey HSD test at the 5% level.
