# Supplementary material for: Short-Chain and Unsaturated Fatty Acids Increase Sequentially From the Lag Phase During Cold Growth of Bacillus cereus
Source: Front Microbiol. 2021 Jul 22;12:694757. doi: 10.3389/fmicb.2021.694757 (PMC8339379; doi:10.3389/fmicb.2021.694757)
Supplement: Supplementary file 1 [file Data_Sheet_1.ZIP › Figure S5.pdf]

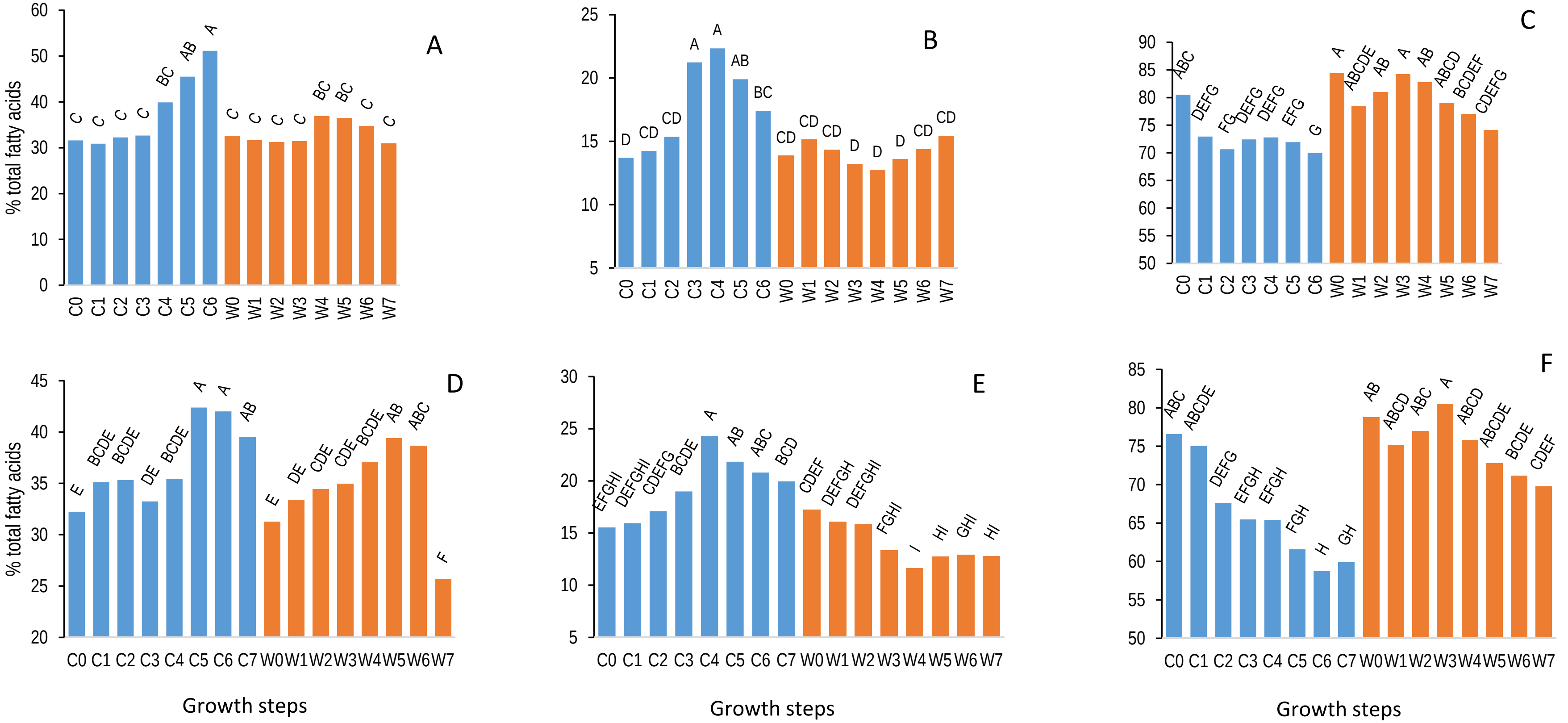

Figure S5a - Changes for ATCC 14579^{T} (A, B, C) and MM3 (D, E, F) strains of *B. cereus* at cold (C0-7, blue bars) and warm (W0-7, orange bars) temperatures of relative abundance of total unsaturated fatty acids (A, D), of fatty acids with less than 15 carbons (B, E),) and of total branched chain fatty acids (C, F). Growth steps C0-6 for ATCC 14579^{T} correspond to 0, 3, 7, 24, 48, 72, 96 h at 12°C and C0-7 for MM3 to 0, 3, 5, 7, 24, 48, 72, 96 h at 10°C. W0-7 correspond for both strains to 0, 0.5, 1, 2, 4, 6, 7, 24 h at 30°C. Results are the mean of three independent experiments. Bars sharing the same letter are not significantly different according to Tukey HSD test at the 5% level.

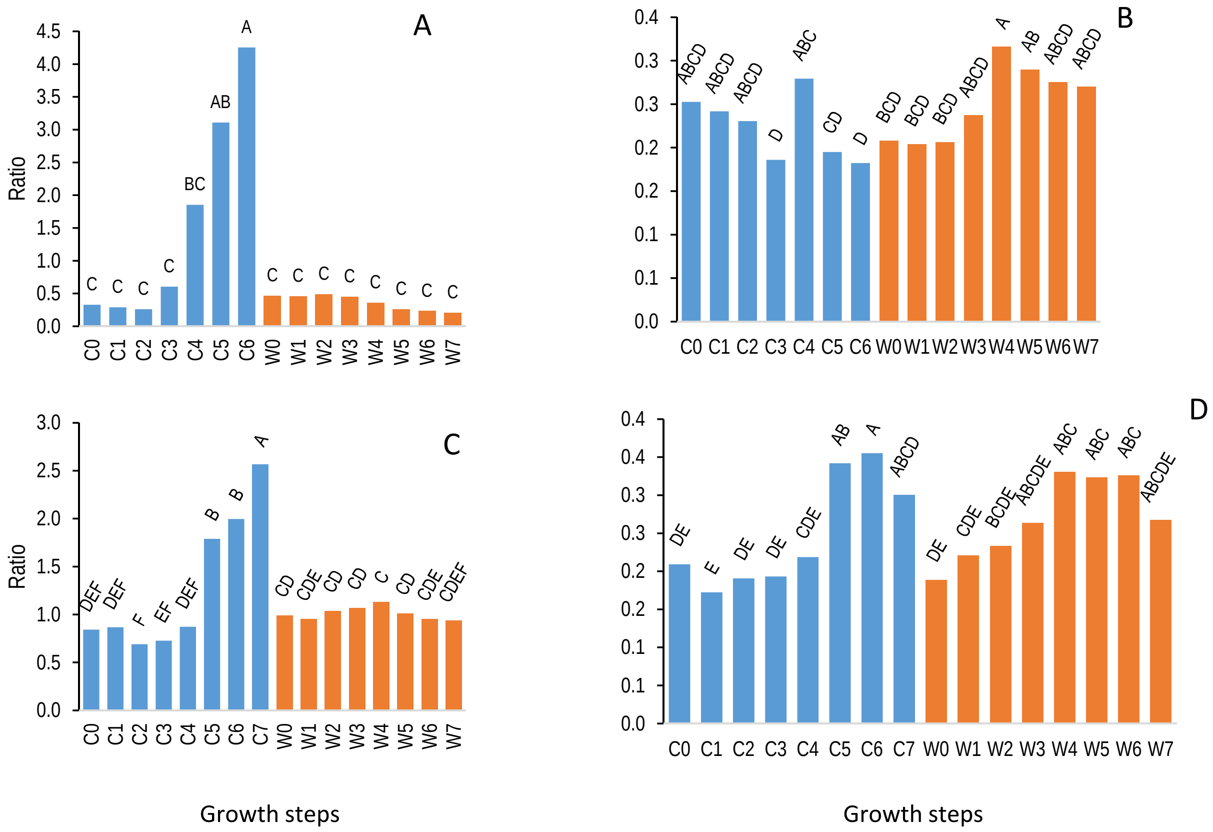

Figure S5b - Changes for ATCC 14579^T (A, B) and MM3 (C, D) strains of *B. cereus* at cold (C0-7, blue bars) and warm (W0-7, orange bars) temperatures of ratio Δ^5 to Δ^{10} mono-unsaturated fatty acids (A, C) and of ratio anteiso to iso branched chain fatty acids (B, D). Growth steps C0-6 for ATCC 14579^T correspond to 0, 3, 7, 24, 48, 72, 96 h at 12°C and C0-7 for MM3 to 0, 3, 5, 7, 24, 48, 72, 96 h at 10°C. W0-7 correspond for both strains to 0, 0.5, 1, 2, 4, 6, 7, 24 h at 30°C. Results are the mean of three independent experiments. Bars sharing the same letter are not significantly different according to Tukey HSD test at the 5% level.
