# Supplementary material for: Short-Chain and Unsaturated Fatty Acids Increase Sequentially From the Lag Phase During Cold Growth of Bacillus cereus
Source: Front Microbiol. 2021 Jul 22;12:694757. doi: 10.3389/fmicb.2021.694757 (PMC8339379; doi:10.3389/fmicb.2021.694757)
Supplement: Supplementary file 1 [file Data_Sheet_1.ZIP › Figure S1.pdf]

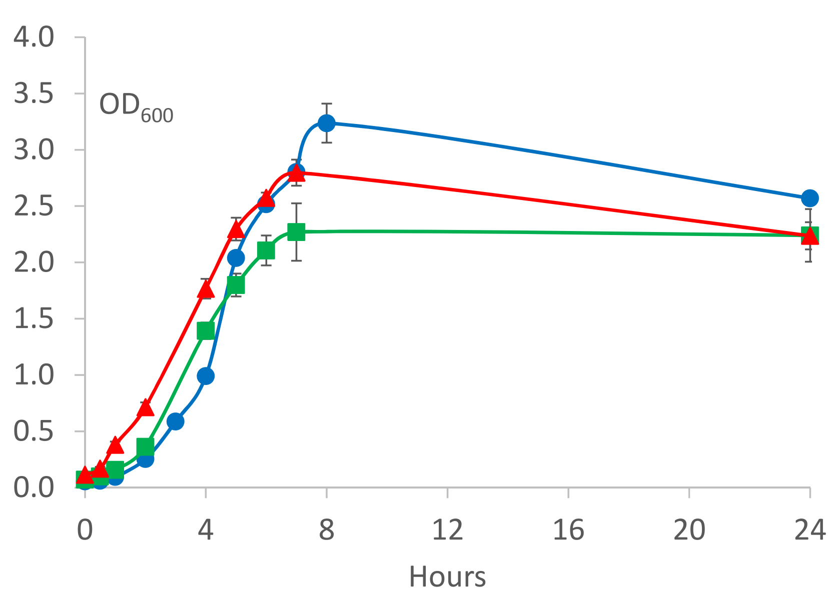

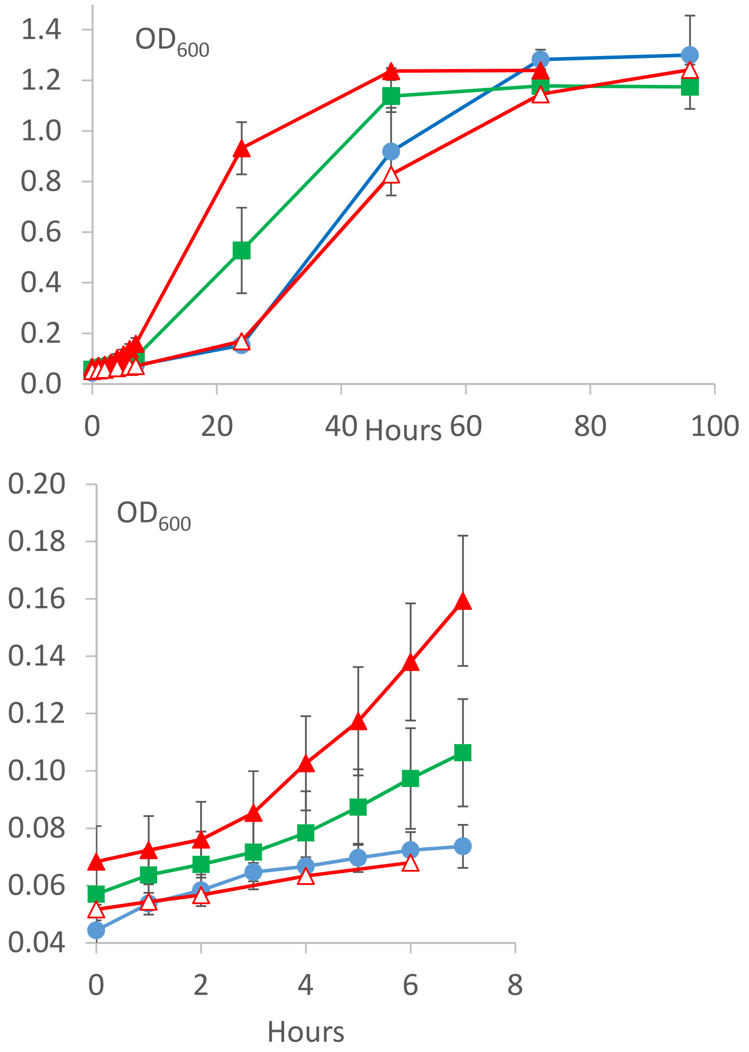

Figure S1a - Growth of the three strains of *B. cereus* ATCC 10876 (green squares), ATCC 14579^{T} (blue circles) and MM3 (red triangles) at warm (left-hand panel) and cold (right-hand panels) temperatures, in flask used to produce cells for fatty acids analysis. Warm temperature was 30 °C for all strains. Cold temperatures were 12 °C (close symbols) and 10 °C for MM3 only (open symbols).
At cold temperatures, growth during the first 7 hours are detailed in the lower right hand panel. Error bars represent standard deviation (n = 3),

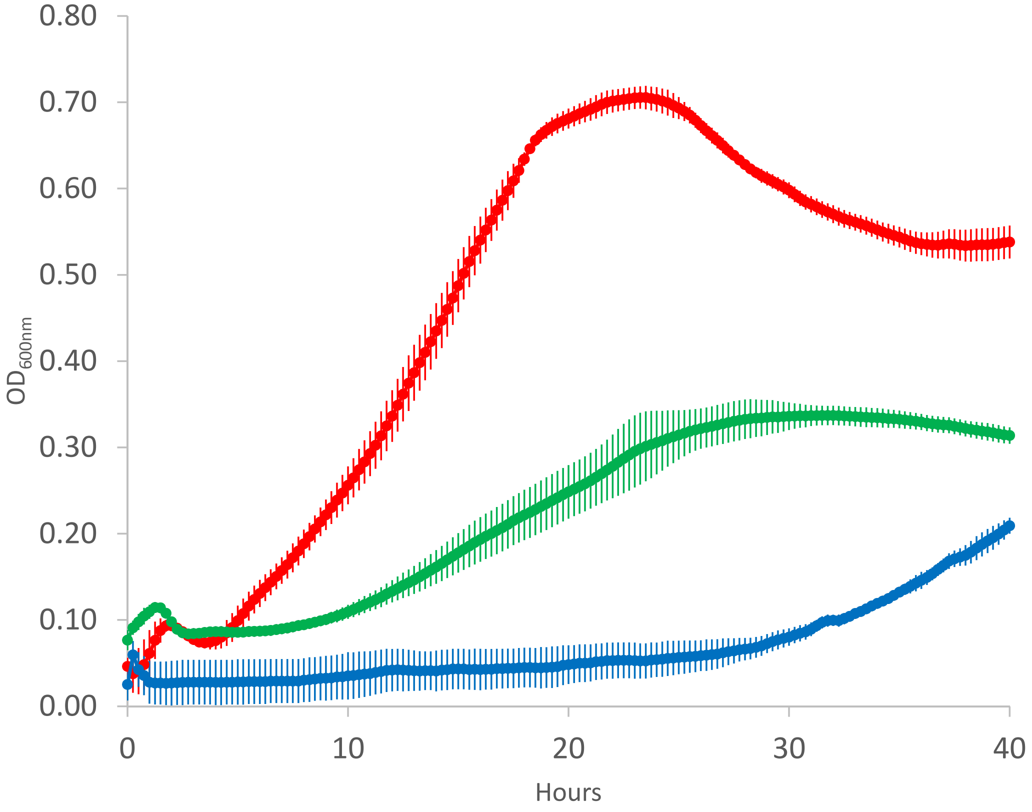

Figure S1b – Growth of *B. cereus* strains (upper to lower) MM3 (red), ATCC 10876 (green) and ATCC 14579^{T} (blue) at 12°C in a microtiter plate. Results are mean of three independant experiments. Vertical bars represent standard deviations.
